# A1 protein free milk benefits mood and subjective cognition in free-living Australian adults: a pragmatic, exploratory, open label randomised controlled trial

**DOI:** 10.3389/fnut.2025.1579986

**Published:** 2025-06-03

**Authors:** Carlene Starck, Michelle Blumfield, Peter Petocz, Emily Duve, Lucy Downey, Kylie Abbott, Flavia Fayet-Moore

**Affiliations:** ^1^FOODiQ Global, Sydney, NSW, Australia; ^2^Department of Statistics, Macquarie University, Sydney, NSW, Australia; ^3^School of Environmental and Life Sciences, The University of Newcastle, Callaghan, NSW, Australia

**Keywords:** A1 protein free milk, brain health, mood, mental health, gastrointestinal microbiome, β-casein, gut-brain axis, pragmatic clinical trial

## Abstract

**Background:**

Adverse effects of milk containing A1-type β-casein on digestion, immune response, and cognition have been identified in milk-intolerant individuals, but health effects in healthy individuals without symptoms of milk intolerance are yet to be examined.

**Objective:**

The objective was to explore the impact of reducing A1 type β-casein intake via switching milk type from conventional A1/A2-type β-casein milk (A1/A2 milk) to A1-type β-casein protein free milk (A1PF) on brain, immune response, gastrointestinal, and skin (BIGS) outcomes in a real-world setting.

**Methods:**

An open-label, pragmatic, exploratory randomised controlled trial was conducted in 997 healthy, free-living Australian older adolescents and adults (16–65 years) who regularly consume A1/A2 protein-containing milk and milk products. Participants were randomised into two groups, to consume ≥250 mL/day of A1/A2 milk (control) or to switch to ≥250 mL/day of A1PF milk (intervention) for 28 days, while continuing to follow their usual diet (including up to 1 serve a day of A1/A2 dairy products). A sub-group of 265 participants conducted stool, saliva and cognitive testing on days 0 and 28. All participants completed subjective questionnaires on days 0, 14, and 28.

**Results:**

No differences in gut microbiome composition, alpha-diversity, or function were found by switching milk type. After switching to A1PF milk, a small increase in stool consistency was reported (−0.16, *p* = 0.007), and females experienced a marginal reduction in gastrointestinal symptoms (*p* = 0.015) and improved subjective cognition (*p* = 0.03). Switching to A1PF milk reduced anxiety (−0.61; *p* = 0.002), depression (−0.56; *p* = 0.023), stress (−0.70, *p* = 0.012) and fatigue (*p* = 0.001; females only), compared to drinking A1/A2 milk, with stronger effects in females. No consistent effects on markers of immune response or skin health were identified.

**Conclusion:**

Switching from conventional A1/A2 milk to A1PF milk may benefit mood and subjective cognition, particularly in females, without the need for complete elimination of A1 β-casein from the diet. Further investigations are warranted.

**Clinical trial registration:**

https://www.anzctr.org.au/Trial/Registration/TrialReview.aspx?id=385966, identifier ACTRN12623000628640.

## Introduction

Cows’ milk is consumed worldwide, contributes a large proportion of nutrients to the diet and has been shown to have several health benefits (1). However, its consumption has been associated with gastrointestinal (GI) discomfort including bloating, and changes in stool consistency, and inflammation (2–4), collectively known as milk intolerance. While mostly attributed to an inability to digest and absorb the lactose in milk, the presence of the A1-type β-casein milk protein can also contribute to milk intolerance (5) and has been suggested to play a role in secondary lactose intolerance (4). As many individuals who self-report lactose intolerance do not have clinical lactose malabsorption (2), it has been proposed that A1-type β-casein intolerance is a common cause of digestive issues experienced following cows’ milk consumption (4, 5). While there are currently 15 known variants of β-casein, these may be categorised into one of two major types, based on the presence of as amino acid substitution at position 67 of the protein sequence; these are A1-type (histidine) and A2-type (proline) (6). In addition to GI issues, milk intolerance has also been suggested to play a role in skin conditions (e.g., dermatitis), immune responses (e.g., asthma), and impaired brain function (e.g., cognitive decline) (5, 7–9). These outcomes can occur independently of GI discomfort, and it is possible that in the absence of GI symptoms, individuals experiencing sub-clinical skin, immune, and brain-related symptoms may find relief when conventional cows’ milk containing A1-type β-casein is reduced in the diet. While A1-type β-casein protein free milk (A1PF) has shown digestive, inflammatory, antioxidant, and some cognitive benefits in participants with reported milk intolerance compared to conventional milk (4, 10–15), it is not known if this effect is unique to milk-intolerant individuals. Understanding the effect of reducing A1-type β-casein protein consumption among healthy individuals by switching their milk type in a real-world setting is required to support dietary advice for all populations.

The adverse health effects of A1-type β-casein may be in part due to the gut-brain axis, such as communication between short chain fatty acids (SCFAs), produced by the gut microbiota, and peripheral body systems (5). There is an established link between diet and gut health via the microbiome, and dysbiosis has been suggested to play a key role in food insensitivities and digestive symptoms (16), as well as poor cognitive function, mood disorders, reduced mental health, weakened immune function, inflammatory skin issues, oxidative stress, and a decreased quality of life (17–22). The microbiome communicates with body systems through a variety of mechanisms including short-chain fatty acids (SCFAs) produced as fermentation by-products by the gut microbiota, as well as both microbe and host-derived hormones, immune molecules, and others (23). In rodents, A1PF milk was found to have benefits for microbiome composition and diversity compared to conventional milk, although effects on levels of SCFAs were inconsistent (24, 25). In humans, evidence is limited, with no data comparing the effects of milks differing in β-casein composition on the microbiome identified to date (5). However, an increase in total faecal SCFAs with A1PF versus conventional milk was observed in people with self-reported milk intolerance (13). Research is required to further understand the effects of milk containing different β-casein profiles on the gut-brain axis.

The majority of interventional research evaluating the relationship between A1 and A2 type β-casein proteins in humans has been carried out via randomised controlled trials (RCTs). These trials represent a high level of evidence and provide the ability to determine a causal relationship under optimal conditions due to being tightly controlled. As this level of control may not be representative of free-living individuals, additional studies are often needed to understand the application of findings to real-world settings. Such additional studies include the pragmatic RCT, which, onducted in free-living individuals and often with an open-label design, maintains randomisation to reduce bias whilst increasing external validity, providing high quality evidence that is generalisable to the population (26). There is a need for pragmatic interventional research to fully understand the differential effect of A1 and A2 type β-casein proteins on human health in real-world conditions, where an individual may choose to simply switch from conventional milk to A1PF milk whilst maintaining an otherwise normal diet that includes other sources of A1 β-casein.

The BIGS (Brain-Immune-Gut-Skin) Trial is an exploratory investigation into the impact of reducing A1-type β-casein intake via switching milk type from conventional milk containing both A1 and A2 β-casein (CON milk) to milk without the A1-type β-casein protein (A1PF milk), on brain, immune, gut, and skin-related outcomes in healthy, free-living older adolescents and adults in a real-world setting. The primary outcomes were gut microbiota composition and diversity, and measures of GI comfort. Secondary outcomes included immune response (incidence of infection or illness and inflammatory markers), skin health, brain health (cognitive function and mental health-related outcomes) and quality of life.

## Methods

### Study design

**Figure 1:**
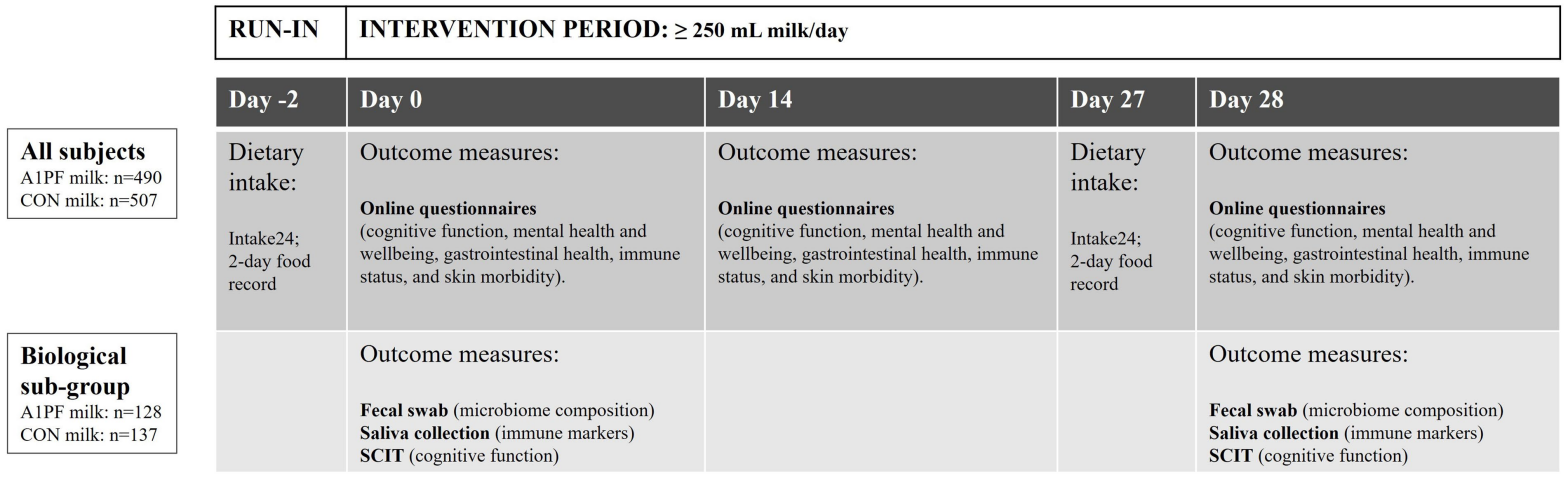
BIGS Trial study design. A 2-day run-in period was followed by a 28-day intervention period in which participants consumed at least 250 mL/day of either A1 protein-free (A1PF) milk or conventional (CON) milk, with up to one serve per day of conventional (A1/A2) dairy products (e.g., cheese, yoghurt). All participants completed a dietary intake assessment (Intake24) and online questionnaires, while the biological subgroup also completed faecal and saliva sampling, as well as the Subtle Cognitive Impairment Test (SCIT) (52). CON milk is milk containing both A1 and A2 β-casein proteins. A1PF milk contains A2 β-casein only.

The BIGS Trial is an Australia-wide open-label mixed methods, pragmatic parallel group RCT that took place over a 28-day period between June 2023 and November 2023. The primary objective was to compare the effect of switching from drinking conventional milk containing both A1 and A2 type β-caseins (CON milk) to milk containing only the A2 type β-casein (intervention, A1PF milk) on gut microbiome composition and diversity as a primary outcome, as well as immune, brain, and skin-related secondary outcomes. A decentralised Australia-wide framework was chosen to encourage a representative sample of the Australian older adolescent and adult population. The study included a sub-group of participants to collect biological samples for objective outcomes including microbiome composition and diversity, GI inflammation, and salivary markers of systemic immune function and inflammation (Figure 1).

Sample size estimates for the total study group were calculated based on the primary subjective outcome measure of GI function, assessed via the Gastrointestinal Symptoms Rating Scale (GSRS) (27). Using available RCT data, a sample size of *n* = 352 per arm was calculated to provide 80% power to detect a significant effect (*p* < 0.05), allowing for 20% attrition (28). No RCTs employing GSRS for food intolerance, nor pragmatic RCTs utilising GSRS were identified and previous observational studies using GSRS to measure GI symptoms have reported sample sizes up to *n* = 1,158 (27, 29). For this study, the sample size of 500 participants per group (*n* = 1,000 participants in total) was selected to allow for non-compliance and to support the collection of population-representative data. Sample size calculations for the sub-group of participants that completed objective biological outcome markers were based on gut microbiome composition using sample size data provided by Microba Life Sciences (30), aiming to detect a small to moderate effect size with 80% power and 20% attrition. The required estimate was *n* = 260 participants (*n* = 130 per arm), including allowance for non-compliance.

An open label study design was selected to align with the pragmatic nature of the trial, as well as ensure a logistical approach to supplying milk to the large number of participants across Australia. To maintain the integrity of the trial and reliability of the results obtained, blinding was included wherever possible to do so, primarily for those involved in data collection and analysis, as well as during randomisation and allocation concealment (described below).

Ethical approval was obtained from the Bellberry Human Research Ethics Committee (HREC2023-04-459-A-1). The trial was prospectively registered with the Australian New Zealand Clinical Trials Registry (ACTRN12623000628640). All participants provided written informed consent and could withdraw at any time without disadvantage. While participants were provided with general information about the study design and intended outcomes as per ethics committee requirements, information about the study hypotheses was not disclosed.

### Participants

A total of *n* = 1,069 participants were recruited for the primary cohort, with a sub-group of *n* = 282 allocated to biological sampling. Any participant who withdrew during the run-in period was replaced. Participants were recruited via a clinical trial recruitment agency and paid social media advertising. Eligibility criteria were designed to enable the recruitment of a sample that was representative of the broader healthy Australian population, and to balance controlling inter-rater variation against recruitment feasibility, with no compromise to participant safety. Participants were required to reside in Australia, be 16–65 years of age, have a BMI from 18.5 to 35 kg/m^2^, read and speak English, be frequent consumers of conventional milk, defined as consumption at least 5 times per week, and be willing to consume no more than one serve of conventional (A1/A2) dairy products per day (equivalent to approximately 0.3–4 g A1 β-casein protein/serve, depending on type of dairy product). Participants were excluded if they were unable to provide informed consent, were diagnosed with a chronic disease or mental health condition that was unable to be or not being effectively managed, were currently pregnant or breastfeeding, undertook hospitalisation or antibiotic use in the past 4 weeks, had changed oral contraception within past 3-months or planned to occur within the study period, or were currently participating in another biomedical or medical study. Eligibility for inclusion within the biological subgroup was dependent on a participant residing in a postcode that enabled timely collection and delivery of biological samples for analysis. A computer-generated randomisation sequence was used to assign participants to either the intervention or control group. The sequence was generated in blocks of 8, with no stratification. Randomisation and allocation concealment was performed by a research scientist (KA) who was not involved in any other aspect of intervention delivery, data collection, or data analysis. Participants enrolled in the study were invited to provide biological samples until the required sample size for each of the intervention and control groups was reached.

### Intervention

Following enrolment and during a 2-day run-in period, participants completed a 24-h food recall on each day (Figure 1). Participants were required to maintain their existing diet and any medication and supplement use for all trial periods (i.e., run-in, wash out, intervention). From days 0 to 28, participants were required to consume at least 250 mL of fresh, 4% fat cows’ milk per day. The intervention group was asked to consume Cows’ milk free of the A1-type β-casein protein (A1 protein free milk; A1PF milk), while the control group consumed conventional milk containing both A1 and A2 β-casein proteins (CON milk). Participants purchased the milk using Coles or Woolworths supermarket gift vouchers provided weekly by the research team, at a minimum of 2 × 1 L bottles per week, equivalent to 8 L over 28 days. Additional milk was available to all participants, based on their usual daily milk intake. No maximum level of consumption was set. To maintain the pragmatic nature of the trial, participants were not provided with specific instructions on how to consume the milk, other than to ensure they consumed the minimum amount of 250 mL/day. Participants were instructed to maintain their existing diet, restricting additional conventional (A1/A2) dairy product intake (such as cheese, yoghurt, and ice-cream) to no more than one serve per day. This study was not a full exclusion of A1 β-casein protein and participants could consume additional A1 β-casein protein from other dairy products (approximately 0.3–4 g/serve, depending on type of dairy product). This trial was not designed to test the presence versus absence of A1 β-casein on BIGS outcomes, but rather the effect of a reduction in intake of A1 β-casein, via switching from conventional milk to A1PF milk. Compliance with the intervention was assessed twice a week.

### Data collection

Data collected are provided in Table 1. All participants completed validated subjective questionnaires online at three time points: baseline (day 0), day 14, and day 28 (trial end). Objective biological sampling was completed at home in a sub-group of participants on days 0 and 28.

**Table 1:** Subjective and objective measures used to assess the impact of conventional vs A1 protein free milk on BIGS outcomes.

Outcome	Subjective measures	Objective measures
Brain	Cognitive function: PROMIS-SF Cognitive Function 6a Mental health and wellbeing: Depression, Anxiety and Stress Scale-21 Items (DASS 21) PROMIS-SF Fatigue 4 World Health Organisation – Five Well-being Index (WHO-5) EuroQoL visual analogue scale (EQ-VAS) PROMIS-SF Sleep Disturbance 6a	Cognitive function: Subtle Cognitive Impairment Test (SCIT)
Immune	Immune Status Questionnaire (ISQ) Respiratory Symptoms Questionnaire (RSQ)	Salivary markers: Cortisol IL-6 IL-1b Secretory IgA (sIgA) TNF-alpha Total glutathione
Gastrointestinal	Gastrointestinal Symptoms Rating Scale (GSRS) Bristol Stool Chart (BSC)	Microbiome composition Microbiome diversity Microbiome functions Faecal calprotectin
Skin	Skin morbidity questionnaire	

BIGS, brain, immune function, gastrointestinal function, skin health; IL, interleukin.

#### Dietary data

Each participant was asked to report their dietary intake from midnight to midnight over 2 days prior to both study commencement and at completion using INTAKE24, an online, self-administered computerised multiple-pass 24-h recall system (Intake24).^1^ INTAKE24 is a validated tool that includes questions and photographs to estimate food and beverage consumption (31, 32).

#### Questionnaire data

Online questionnaires were completed by participants via the online survey platform QuestionPro to assess subjective outcomes. A full description of the subjective questionnaires and data collected are provided in Supplementary Table 1. The tests included the GSRS, the Bristol Stool Chart (BSC), Immune Status Questionnaire (ISQ), PROMIS-SF Cognitive Function 6a, PROMIS-SF Fatigue 4, Skin morbidity questionnaire, Depression, Anxiety and Stress Scale-21 Items (DASS 21), Respiratory Symptoms Questionnaire (RSQ), World Health Organisation – Five Well-being Index (WHO-5), EuroQoL visual analogue scale (EQ-VAS), and PROMIS-SF Sleep Disturbance 6a. Adverse events were captured within each survey, as appropriate. To avoid survey fatigue, the questionnaire length, number, and complexity of questions and open-ended question type were considered.

#### Faecal data

*Sample collection.* Faecal samples were self-collected by participants using commercial collection kits provided by Microba Life Sciences Ltd. (Brisbane, Australia). To collect the faecal sample, participants emptied their bladder and then collected a stool sample. Samples were placed into a pre-labelled biological specimen bag and sealed in the pre-addressed envelope provided and posted via express mail to the laboratory for analyses. The sample collection kit allowed for faecal samples to remain stable for 60 days at room temperature.

*Sample processing and quality control.* Microbiome samples were processed and analysed by Microba Life Sciences Ltd. Microba laboratory operations follow ISO15189 guidelines set by National Authority of Testing Australia (NATA). Samples were visually inspected for any quality issues (e.g., broken tubes, overloading, under loading), stored at −80°C, and collated into batches for processing.

*DNA extraction and metagenomic sequencing.* DNA extraction was performed using the DNeasy 96 PowerSoil Pro QIAcube HT Kit (Qiagen 47021), with proprietary workflow optimisation steps on the QIAcube HT DNA extraction system (Qiagen 9001793). Microbiome libraries were prepared with an optimised high-throughput format of the manufacturer’s protocol using the Illumina DNA Prep (M) Tagmentation Kit (Illumina, 20018705) with IDT for Illumina DNA/RNA UD Index Sets A-D (Illumina 20027213-16). Libraries were pooled at equimolar amounts to create a sequencing pool. The pool was quantified, and quality control was performed with gel analysis, qubit measurement and qPCR. The library was prepared for sequencing on the NovaSeq6000 (Illumina) using NovaSeq6000, v1.5 reagents, and 2 × 150 bp paired-end chemistry in the Microba laboratory according to the manufacturer’s protocol. Pools were sequenced to a target depth of 3 Gb per sample with a minimum of 2 Gb. Data was quality controlled to remove low quality sequences and human DNA.

*Bioinformatics analysis.* Microbial data generated from the metagenomic sequencing of the study samples was processed using four key proprietary bioinformatic systems from Microba Life Sciences Ltd.: the Microba Genome Database (MGDB), the Microba Community Profiler (MCP), the Microba Genes Database (MGENES), and the Microba Genes and pathway Profiler (MGPP). Species-level genome-based abundances were calculated using the MCPv2, which utilises a curated set of genome sequences from the public domain and Microba proprietary data (MGDB v2.0.0). Species in the prokaryote profile were assigned taxonomy using the systematic genome-based Genome Taxonomy Database.^2^ Relative abundances of genes were calculated using Microba’s MGENES database, which contains >50 million gene clusters. Genes were annotated using UniProt, Enzyme Commission (EC), and Transporter Classification Database (TCDB) annotations, where possible. The MGPP estimates counts of genes per sample and per species for all MGENES, EC, and TCDB entries. This functional data was also used to quantify higher level metabolic pathways utilising pathway annotations from MetaCyc.

*Faecal calprotectin.* Calprotectin levels were measured quantitatively by Microba Life Sciences Ltd. using microbiome DNA-extracted samples that had been stored at −20°C until processing. Testing was carried out via the Immunodiagnostik enzyme-bound immunosorbent assay (ELISA) kit (IDK Calprotectin (MPR 8/14)), as per the manufacturer’s instructions. The assay utilises the two-site sandwich technique with two selected monoclonal antibodies that bind to human calprotectin. The peroxidase in the final complex (capture antibody—human calprotectin—peroxidase conjugate) undergoes colorimetric reaction with the substrate tetramethylbenzidine (TMB) to produce a final yellow colour, detected at 450 nm, which is directly proportional to the calprotectin concentration of the sample. Input volume for ELISA processing was determined based on a required ELISA input of 15 mg, with a 5,200-fold total dilution factor used. Measurements categorised as within a normal range (<50 μg/mL), borderline (50–100 μg/mL), or high/outside of normal range (>100 μg/mL).

#### Saliva data

*Sample collection.* Saliva samples were self-collected by participants 1 h after waking in the morning using collection kits provided by Stratech Scientific. Prior to sample collection, participants were asked to: (i) avoid food, drinks or brushing their teeth for 30 min prior to collection, (ii) stay in a calm environment for 30 min prior to collection, and (iii) rinse their mouth thoroughly with water 10 min prior to collection. Participants placed a SalivaBio Oral Swab (Salimetrics LLC, Carlsbad, USA) under the tongue for 60 s to measure sub-lingual salivary IgA. SalivaBio oral swabs are validated for the measurement of multiple salivary analytes (33). Participants also collected two passive drool samples for all other markers. After collection, samples were placed in a standard household freezer (−18 to −23°C) until samples were collected via courier for dry ice transportation to the laboratory for analysis.

*Sample processing and quality control.* Saliva samples were processed and assayed by Stratech Scientific (Mona Vale, Australia). The collected saliva samples were stored frozen at −20°C until assay and were limited to two freeze thaw cycles. On the day of assay, samples were thawed and analysed using commercially available kits according to the manufacturer’s instructions: cortisol, IL-6, IL-1β and secretory IgA (sIgA) (Salimetrics, USA) and TNF-alpha (Abcam, UK) were analysed via ELISA. Total glutathione was analysed via colorimetric assay (OxiSelect™ Total Glutathione Assay Kit, Cell Biolabs, Inc., USA). Thawed samples were centrifuged at 1,500×*g* for 15 min to collect clear saliva and this saliva was used without further processing for all assays. All samples were brought to room temperature before adding to the assay wells and all samples were analysed in duplicate.

#### Objective cognitive data

Cognition was measured objectively via the validated online Subtle Cognitive impairment Test (SCIT)^3^ (34). The “H” version (more challenging, appropriate for those aged 15–70 years) of the SCIT was used (34). During the SCIT, participants were briefly shown a pair of lines on a computer screen and asked to indicate which side was the shortest by pressing a keyboard button. This process was repeated many times while the speed of response and number of errors made was recorded. SCIT scores were calculated based on three continuous measures: 1. Speed of response to a stimulus (measured to the nearest millisecond); 2. the percentage of incorrect responses (mean error rate); 3. The number of times a participant fails to respond to a stimulus (number of Timed Outs). The SCIT test utilises very quick durations of a stimulus that may not be consciously recognisable but are available to subcortical parts of the brain and the visual cortex (Head scores), as well as durations of 80 ms or more, where the stimulus is displayed for long enough that it reaches conscious awareness (Tail scores) (35). SCIT scores can be used for two purposes: to track small changes in cognitive performance over time; or, as a one-off measure to assess brain function by comparison to normative measures (35). The former purpose was applied to the BIGS trial. The SCIT was selected for its sensitivity to mild cognitive impairment as well as its association with systemic inflammation and sleep quality (34). The SCIT was limited to the nested biological subgroup. Subjective cognitive function was assessed by all participants via questionnaire (see Methods section 4.2).

### Statistical analysis

#### Microbial data

Microbial data were analysed using R software version 3.4. Differentially abundant taxa (species, genera, family, and phyla) and functions (EC, TCDB, MetaCyc) were identified by linear mixed effect regression (LMER) of square root (sqrt) and centre-log ratio (clr) transformed relative abundances. The model included random effects for participant ID and fixed effects for milk type (CON milk and A1PF milk), time, and milk type over the 28-day trial. Changes occurring during the course of the trial within each milk type arm were also determined. For each dataset, transformed data were statistically compared across the study groups and computed *p* values were corrected for multiple testing (False Discovery Rate, FDR) using the Benjamini-Hochberg procedure. Rare features present in less than 10 samples and low abundance features with a maximum sample count of less than 100 for taxonomic data and less than 2 for functional analyses were excluded. *p* values < 0.05 after correction were considered statistically significant. Level of significance for each outcome variable was determined based on ranking by *p*-value across all applied univariate tests. Data were analysed both with and without covariate adjustment, where covariates were age, sex, dietary fibre intake (g/day), usual milk intake (mL/day), and presence of any health condition risk factors (e.g., high cholesterol, high blood pressure, migraines). Shannon diversity and richness were compared using LMER, including participant ID as a random effect and milk type, time, and milk type over time as fixed effects. Data was rarefied to 8,372,956 reads. All microbiome data are presented as means ± SDs, where applicable.

#### Salivary and subjective data

All analyses were performed with IBM SPSS version 29 (New York, USA). Each outcome was analysed using a repeated measures general linear model, with independent variables of milk type, sex, milk type and sex interaction, usual milk intake, and age. Where appropriate, the Greenhouse–Geisser adjustment was made for non-sphericity, determined using Mauchly’s test of sphericity. Due to the number of outcome variables and the potential to overestimate an effect due to the open label nature of the trial, statistical significance was taken as *p* < 0.01, and *p*-values between 0.01 and 0.05 were regarded as marginally significant. Pre- and post-intervention comparisons (day 0 vs. day 28) were conducted via independent samples t-tests for numerical variables or chi-squared tests for categorical variables, with *p* < 0.05 regarded as significant.

## Results

### Participant details

**Figure 2:**
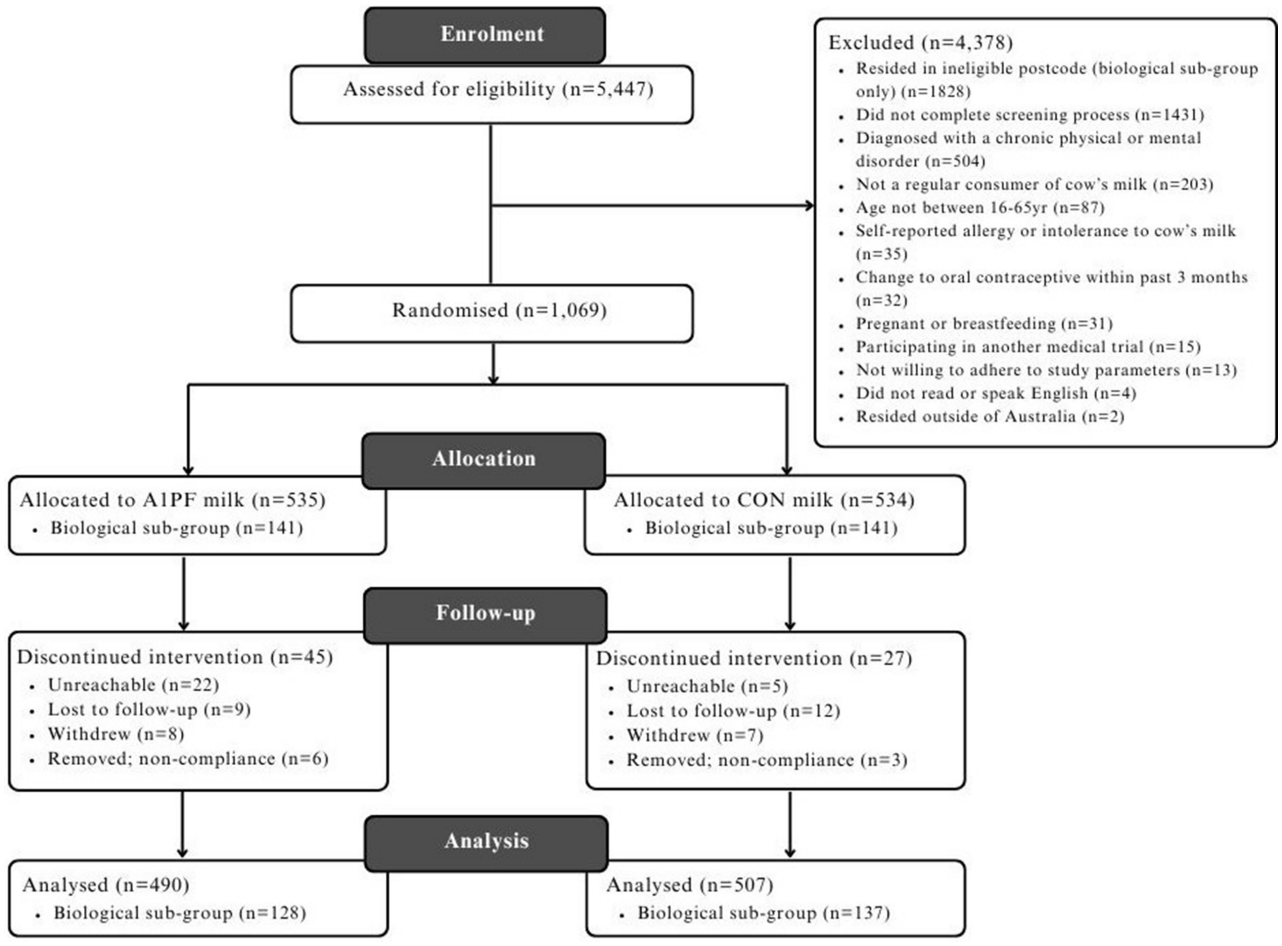
CONSORT diagram detailing the flow of participants through the BIGS Trial. CON milk, conventional milk containing both A1 and A2 β-casein proteins; A1PF milk, milk containing A2 β-casein only.

A CONSORT diagram detailing the study flow is provided in Figure 2. Baseline characteristics of participants are provided in Table 2, with a final sampling size of *n* = 997, with a sub-group of *n* = 265 that completed biological sampling. No differences between study arms were found for the majority of measures, except for usual milk intake (higher intake in CON milk arm for the total group, *p* < 0.001) and employment status (greater proportion of full-time employment in the CON milk arm, and more retired individuals in A1PF milk arm, *p* < 0.05) within the total study group, and age between milk types within the biological sub-group (older individuals in A1PF arm, *p* = 0.028). There was no difference in baseline additional dairy intake between the CON and A1PF study arms.

**Table 2:** Baseline participant data for each study group^1^.

Variable	Total study group		Biological sub-group	
	CON milk	A1PF milk	CON milk	A1PF milk
Participants (*n*)	507	490	137	128
Sex, *n* (%)				
Female	312 (61.5%)	301 (61.4%)	82 (59.9%)	77 (60.2%)
Male	195 (38.5%)	189 (38.6%)	55 (40.1%)	51 (39.8%)
Age^2^, y	34.4 (13.1)	34.2 (13.1)	33.7 (11.3)	36.7 (11.3)*
Education, *n* (%)				
Up to high school	123 (24.5%)	136 (28.3%)	20 (14.7%)	12 (9.4%)
Trade certificate or diploma	82 (16.3%)	78 (16.3%)	17 (12.5%)	28 (21.9%)
Graduate/postgraduate degree	297 (59.1%)	266 (55.4%)	99 (72.8%)	86 (68.7%)
Born in Australia, *n* (%)	329 (65.7%)	304 (62.1%)	85 (63.0%)	74 (57.9%)
Household income, AUD$ *n* (%)				
<$30,000	39 (7.8%)	29 (6.0%)	9 (6.7%)	8 (6.3%)
$30,000–$99,999	125 (25.0%)	142 (29.6%)	30 (22.3%)	31 (24.2%)
$100,000–$199,999	193 (38.5%)	162 (33.7%)	54 (40.0%)	46 (35.9%)
>$200,000	74 (14.8%)	68 (14.1%)	29 (21.4%)	24 (18.8%)
Employment, *n* (%)^3^				
Unemployed	68 (13.6%)	72 (15.0%)	12 (8.9%)	5 (3.9%)
Part time/casual	188 (37.6%)	190 (39.6%)	46 (34.0%)	45 (35.2%)
Full time	234 (46.7%)	201 (41.9%)*	76 (56.3%)	74 (57.8%)
Retired	11 (2.2%)	17 (3.5%)*	1 (0.7%)	4 (3.1%)
Height^2^ (cm)	170 (10)	169 (10)	170 (10)	169 (10)
Weight^2^ (kg)	72.3 (15.6)	70.9 (15.4)	71.0 (15.3)	69.9 (13.9)
BMI^2^ (kg/m^2^)	25.0 (4.5)	24.7 (4.7)	24.6 (4.1)	24.5 (4.0)
Current/former smoker, *n* (%)	84 (16.8%)	64 (13.4%)	20 (14.8%)	21 (16.4%)
Alcohol consumption, *n* (%)				
None	281 (56.3%)	287 (60.2%)	73 (54.1%)	69 (53.9%)
Up to 7 standard drinks/week	156 (31.3%)	146 (30.6%)	43 (31.9%)	46 (35.9%)
Over 7 standard drinks/week	62 (12.4%)	44 (9.2%)	19 (14.1%)	13 (10.2%)
Usual dairy intake, total^2^^,^^3^ (g/day)	553 (521)	506 (610)	491 (405)	537 (690)
Dairy milk^3^	398 (190)	352 (138)**	368 (151)	347 (160)
Other dairy^3^	152 (553)	149 (507)	122 (377)	187 (642)
Health condition^4^, *n* (%)				
Previous diagnosis	211 (41.7%)	211 (43.1%)	58 (42.6%)	48 (37.5%)
No diagnosis	295 (58.3%)	278 (56.9%)	78 (57.4%)	80 (62.5%)
Current medications, *n* (%)	47 (9.4%)	61 (12.8%)	8 (5.9%)	15 (11.7%)
Current supplementation, *n* (%)	156 (31.2%)	157 (32.9%)	43 (31.9%)	49 (38.3%)
Vaccinations (all), *n* (%)				
<3 months ago	63 (12.6%)	42 (8.8%)	22 (16.3%)	11 (8.6%)
3–6 months ago	126 (25.3%)	126 (25.3%)	35 (25.9%)	33 (25.8%)
6–12 months ago	112 (22.4%)	106 (22.2%)	30 (22.2%)	28 (21.9%)
>12 months ago	168 (33.7%)	180 (37.7%)	41 (30.4%)	51 (39.8%)
Not vaccinated/no response	30 (6.0%)	21 (4.4%)	7 (5.3%)	5 (3.9%)

1 All participants completed subjective analyses. The biological subgroup also completed biological sampling (stool, saliva, and SCIT).

2 Mean ± SD (all such values). The presence of a statistically significant difference between study arms is depicted as *p < 0.05, and **p < 0.01. Comparisons conducted via independent samples t-tests for numerical variables or chi-squared tests for categorical variables, with *p* < 0.05 regarded as significant.

3 Usual milk intake data was collected specifically. Other dairy intake was obtained from data reported via Intake24 over two days immediately prior to the start of trial and includes milk, cheese, yoghurt, chocolate, and milk-based desserts. The conversion of mL to g for dairy milk assumes that 1 mL = 1 g. Note: Total dairy is only calculated/used for those subjects who gave values for both Dairy milk and Other dairy.

4 Health conditions include chronic issues such as type 2 diabetes, cardiovascular disease, and depression. Individuals with previously diagnosed but currently managed chronic conditions were included in the trial in order to produce a sample representative of the broader healthy Australian population.

std, standard; A1/A2, conventional milk containing both A1 and A2 β-casein proteins (control); A1PF, milk containing A2 β-casein only (intervention).

### Primary health outcomes

#### Gut microbiome

**Figure 3:**
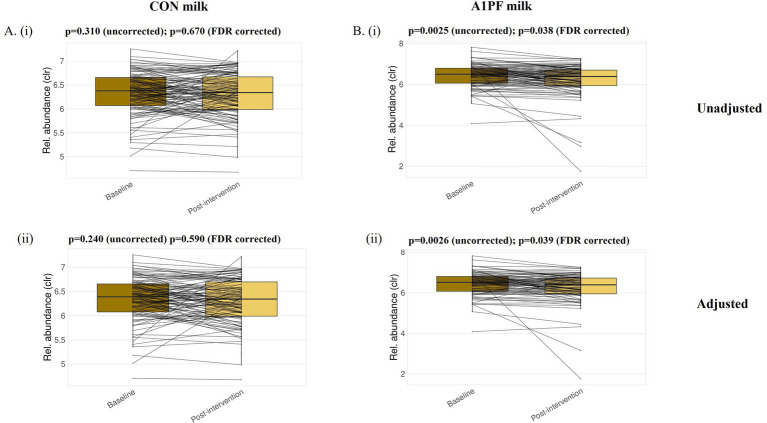
Differential abundance of the Firmicutes A phylum at baseline and post-intervention for each of the conventional (CON) milk **(A)** and A1 protein free (A1PF) milk **(B)** study groups. Analysis was conducted for unadjusted (i) and adjusted (ii) datasets. Significance was taken as *p* < 0.05 after correction for false discovery rate (FDR). CON milk is milk containing both A1 and A2 β-casein proteins. A1PF milk contains A2 β-casein only.

**Figure 4:**
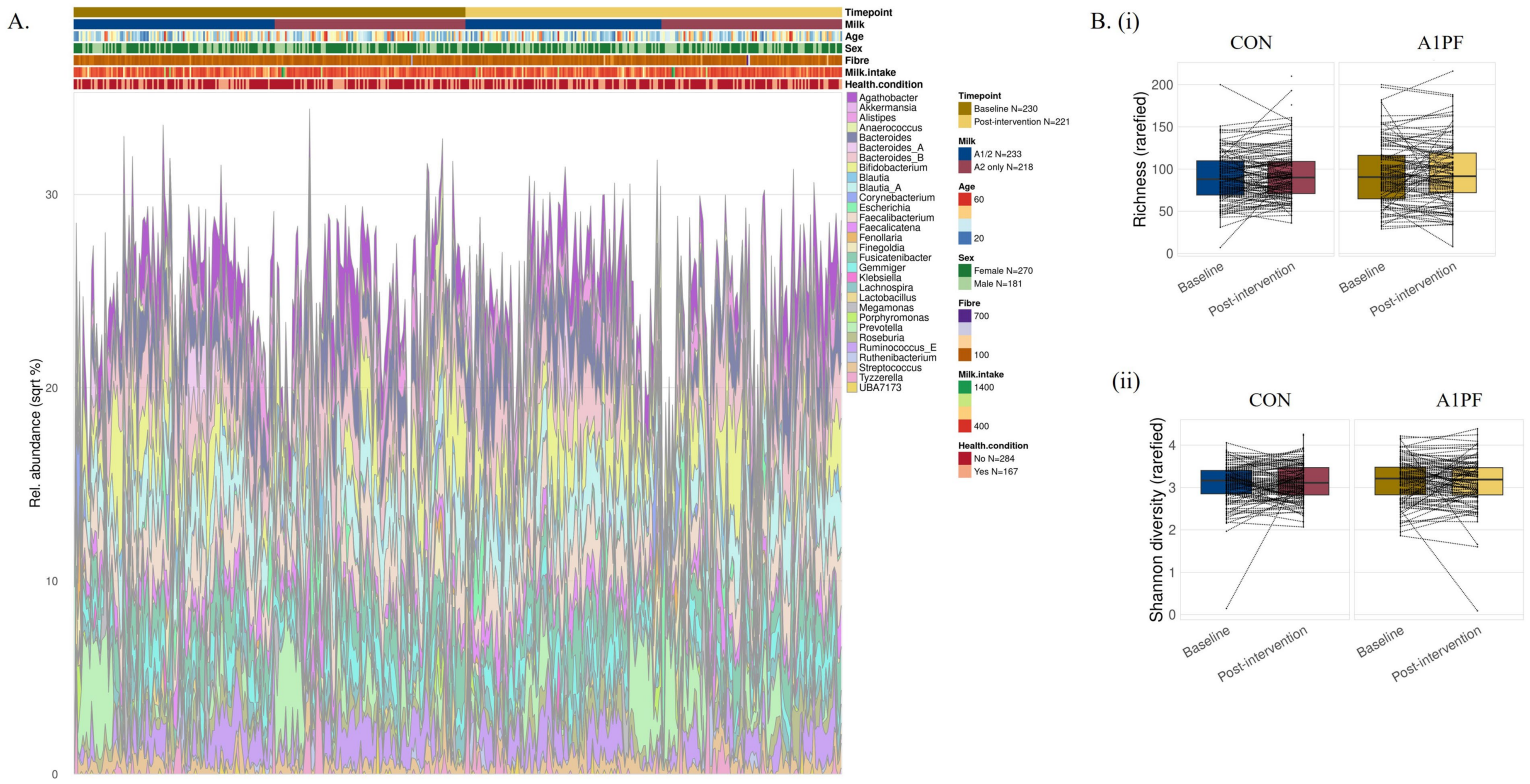
Microbiome composition **(A)** and differential diversity **(B)** at the genus level for conventional (CON) milk compared to A1 protein free (A1PF) milk. Diversity was measured as richness (i) and Shannon diversity (ii). There was no effect of milk type on either outcome. CON milk is milk containing both A1 and A2 β-casein proteins. A1PF milk contains A2 β-casein only.

*Microbiome composition and diversity.* There was no effect of switching to A1PF milk on the composition or diversity of the microbiome in either unadjusted or adjusted (usual milk intake, sex, age, health condition, fibre intake) analyses at any phylogenetic level (species, genus, family, phylum) (full data not shown). While there were significant differences in differential abundance at all phylogenetic levels between milk types in uncorrected analyses (for example, increased *Lactococcus* genus in A1PF milk group compared to CON milk group, *p* < 0.01; full data not shown), this was removed by FDR correction. In analysis of differential abundance over the course of the trial, there was a decrease in the Firmicutes A phylum at the end of the trial for those who switched to A1PFmilk compared to baseline for both unadjusted (*p* = 0.038) and adjusted (*p* = 0.039) clr-transformed data (Figure 3A). No differences were observed within those who continued drinking CON milk (unadjusted *p* = 0.670; adjusted *p* = 0.590) (Figure 3B). There were no differences at the species, genus, or family phylogenetic levels for either milk type group (data not shown). The differential abundance of each phylum (adjusted data) over the course of the trial both within each milk type and between milk types is provided in Supplementary Table 2. The 20 most differentially abundant families, genera, and species between milk types (adjusted data) are shown in Supplementary Table 3. Figure 4 shows the overall composition (A) and diversity (B) at the genus level (adjusted data) over the course of the trial and between milk types, respectively. The predominant genera included *Agathobacter, Bacteriodes, Bifidobacterium, Blautia, Ruminococcus,* and *Prevotella* genera, among others. There were no differences in alpha diversity measured as richness (i; *p* = 0.36) or Shannon index (ii; *p* = 0.22) between milk types; this absence of effect was mimicked at all other phylogenetic levels (data not shown). Similarly, there were no differences in alpha diversity over the course of a trial for either milk type (data not shown).

*Microbiome functional analyses.* Switching to A1PFmilk had no effect on microbiota functions, regardless of the functional database used for analysis. Similarly, there were no functional differences between the start and end of the trial for either milk type. While a number of functions were identified to show significant differential abundance in all analyses, FDR correction removed this significance. The 20 most differentially abundant functions between milk types (adjusted data) are shown in Supplementary Table 4.

#### Gastrointestinal inflammation

There was no effect of switching milk type on levels of faecal calprotectin (*p* = 0.260), nor any effect of sex, age, or usual milk intake (Table 3).

**Table 3:** Gastrointestinal health outcomes for milk free of A1 β-casein (A1PF milk) vs conventional milk containing both A1 and A2 β-caseins (CON milk).

Outcome variable	CON milk^1^			A1PF milk^1^			Difference over time (A1PF-CON)	*p*-values^2^				
	Day 0	Day 14	Day 28	Day 0	Day 14	Day 28		Milk type	Sex	Sex*milk type	Age	Usual milk intake^3^
Subjective questionnaires												
GSRS	1.387 (0.023)	1.475 (0.029)	1.412 (0.025)	1.482 (0.023)	1.554 (0.029)	1.460 (0.025)	−0.046 (0.034)	0.340	0.047*	0.015*	<0.001*	0.019*
BSC	3.617 (0.055)	3.633 (0.056)	3.780 (0.056)	3.631 (0.055)	3.775 (0.056)	3.637 (0.057)	−0.159 (0.091)	0.007*	0.009*	0.720	0.749	0.035*
Stool analysis												
fCal (μg/g)	59.42 (11.87)	ND	58.76 (11.79)	29.99 (12.24)	ND	55.18 (12.16)	25.84 (22.77)	0.260	0.113	0.229	0.914	0.475

1 Values are means (SE).

2 *P*-values were derived using a repeated measures general linear model, with the Greenhouse–Geisser adjustment made for non-sphericity where appropriate. Significance was set at *p* < 0.01 and is indicated in bold with an asterisk. Marginal significance was set between 0.01 and 0.05 and is indicated with an asterisk only.

3 Usual milk intake refers to the mean daily consumption (mL/day) of milk over the course of the trial.

CON milk, milk containing both A1 and A2 β-casein proteins; A1PF, milk containing A2 β-casein only; fCal, faecal calprotectin; ND, not determined.

#### Subjective gastrointestinal health

**Figure 5:**
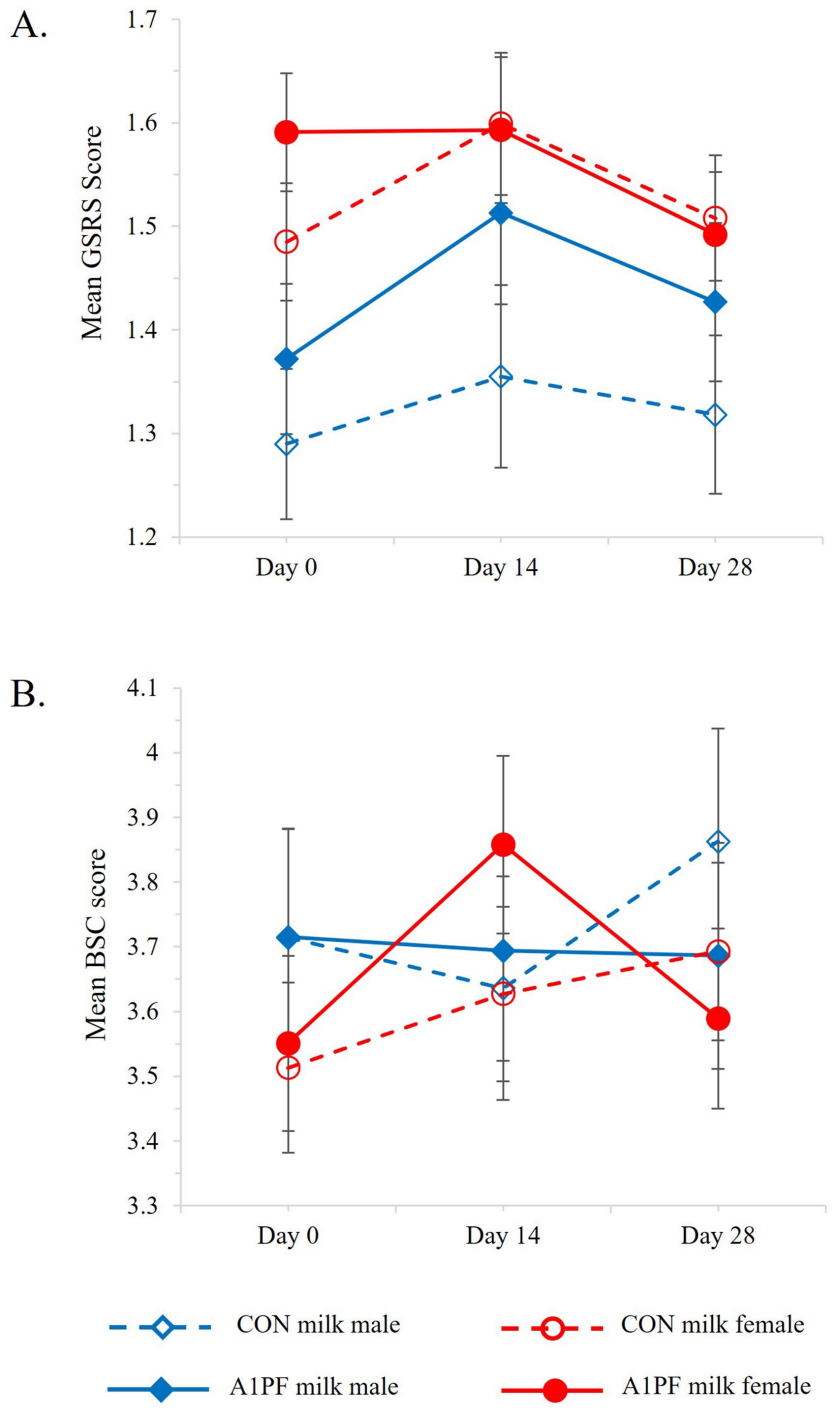
Change in **(A)** gastrointestinal symptoms (GSRS, Gastrointestinal Symptoms Rating Scale) and **(B)** stool consistency (BSC, Bristol Stool Chart) over the course of the trial for CON milk (open marker, hashed lines) compared to A1PF milk (solid marker, solid lines) for males (blue) and females (red). CON milk is milk containing both A1 and A2 β-casein proteins. A1PF milk contains A2 β-casein only.

There was no overall effect of switching milk type on GSRS outcomes (Table 3). However, females who switched to A1PF milk reported reduced GI symptoms compared to those who continued to drink CON milk (*p* = 0.015), and males exhibited reduced GSRS outcomes over the course of the trial compared to females (*p* = 0.047), although significance was marginal in both cases (Figure 5A). Age (*p* < 0.001) and usual milk intake (*p* = 0.019) influenced GSRS outcomes in an inverse manner (data not shown). A switch to A1PF milk affected stool consistency (Table 3), reducing BSC score by 0.16 units (*p* = 0.007) compared to CON milk. Sex influenced stool consistency, with females experiencing an observable increase in BSC (softer stool consistency) at day 14 with the switch to A1PF milk compared to CON milk (*p* = 0.009, Figure 5B); which was reduced to baseline by trial end. Usual milk intake had a marginally significant impact on BSC score (*p* = 0.035), with a small decrease in BSC score with increasing usual milk intake (data not shown).

### Secondary health outcomes

#### Brain

**Figure 6:**
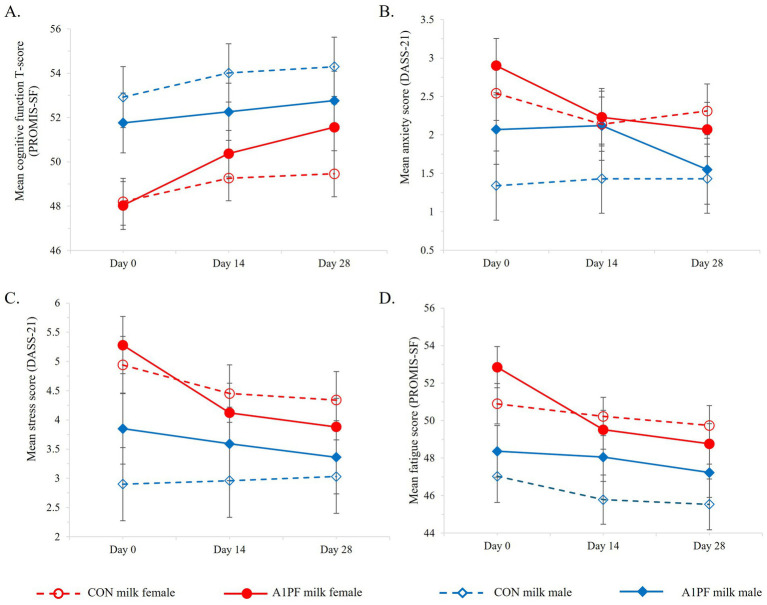
Differential effects of sex on subjective brain-related health outcomes over the course of the trial for CON milk (open marker, hashed lines) compared to A1PF milk (solid marker, solid lines) for males (blue) and females (red), including **(A)** cognitive function; **(B)** anxiety; **(C)** stress; and **(D)** fatigue. CON milk is milk containing both A1 and A2 β-casein proteins. A1PF milk contains A2 β-casein only.

*Cognition.* A switch in milk type had no effect on most cognitive outcomes (Table 4). There were a marginally greater number of errors associated with SCIT performance in the longer exposure durations in those who switched to A1PF milk (SCIT-E_T_ *p* = 0.045), compared to participants drinking CON milk. Females reported a marginal improvement in subjective cognitive function over the trial with A1PF milk compared to CON milk (*p* = 0.030) (Figure 6A).

**Table 4:** Brain related health outcomes for milk containing A2 β-casein only (A1PF milk) vs conventional milk containing both A1 and A2 β-caseins (CON milk).

Outcome variable		CON milk^1^			A1PF milk^1^			Difference over time (A1PF - CON)	*p*-values^2^				
		Day 0	Day 14	Day 28	Day 0	Day 14	Day 28		Milk type	Sex	Sex*milk type	Age	Usual milk intake
Cognition													
Subjective cognitive function T-score^3^		50.58 (0.44)	51.67 (0.42)	51.90 (0.43)	49.88 (0.44)	51.31 (0.42)	52.15 (0.43)	0.94 (0.54)	0.160	0.060	0.030*	0.050	0.620
Cognitive impairment	SCIT-RT_H_	633.2 (11.0)	ND	591.6 (9.1)	623.9 (11.8)	ND	596.2 (9.7)	13.7 (11.4)	0.230	0.220	0.460	0.850	0.920
	SCIT-RT_T_	520.9 (8.4)	ND	499.6 (7.3)	516.4 (9.0)	ND	509.5 (7.8)	14.4 (9.5)	0.140	0.100	0.660	0.430	0.110
	SCIT-E_H_	24.10 (1.45)	ND	21.59 (1.42)	23.12 (1.56)	ND	23.69 (1.53)	3.08 (2.18)	0.160	0.057	0.690	0.580	0.640
	SCIT-E_T_	3.56 (0.63)	ND	2.27 (1.28)	2.83 (0.68)	ND	5.38 (1.37)	3.85 (1.88)	0.045*	0.062	0.720	0.500	0.740
Mental health													
Anxiety^4^		1.93 (0.15)	1.77 (0.15)	1.86 (0.14)	2.49 (0.14)	2.18 (0.15)	1.81 (0.14)	−0.61 (0.19)	0.002**	0.004**	0.750	0.520	0.360
Depression^4^		2.74 (0.19)	2.53 (0.19)	2.55 (0.19)	3.19 (0.19)	2.76 (0.19)	2.44 (0.19)	−0.56 (0.22)	0.022*	0.210	0.370	0.340	0.860
Stress^4^		3.91 (0.20)	3.69 (0.21)	3.67 (0.20)	4.57 (0.20)	3.86 (0.20)	3.63 (0.20)	−0.70 (0.25)	0.012*	<0.001**	0.780	0.250	0.630
Fatigue^5^		48.95 (0.45)	48.00 (0.42)	47.61 (0.43)	50.62 (0.45)	48.79 (0.42)	48.00 (0.43)	−1.28 (0.57)	0.055	0.040*	0.001**	0.070	0.950
Sleep^6^		68.04 (0.38)	68.91 (0.36)	68.57 (0.39)	67.96 (0.38)	69.26 (0.37)	68.66 (0.40)	0.17 (0.60)	0.770	0.310	0.880	0.250	0.530
Quality of Life^7^		76.65 (0.75)	76.66 (0.74)	77.27 (0.71)	74.07 (0.75)	74.61 (0.74)	76.84 (0.71)	2.15 (1.00)	0.070	0.520	0.810	0.063	0.840

1 Values are means (SE).

2 *P*-values were derived using a repeated measures general linear model, with the Greenhouse–Geisser adjustment made for non-sphericity where appropriate. Significance was set at *p* < 0.01 and is indicated in bold with an asterisk. Marginal significance was set between 0.01 and 0.05 and is indicated with an asterisk only.

3 Assessed via the PROMIS-SF Cognitive Function 6a questionnaire.

4 Each assessed as sections of the Depression, Anxiety and Stress Scale-21 Items (DASS 21) questionnaire.

5 Assessed via the PROMIS-SF Fatigue 4 questionnaire.

6 Assessed via the PROMIS-SF Sleep Disturbance 6a questionnaire.

7 Assessed via the World Health Organisation – Five Well-being Index (WHO-5) and EuroQoL visual analogue scale (EQ-VAS) questionnaires.

CON milk, milk containing both A1 and A2 β-casein proteins; A1PF, milk containing A2 β-casein only; RT_H_, head mean response time; E_H_, head mean error rate; ND, not determined; SCIT, Subtle Cognitive Impairment Test; RT_T_, tail mean response time; E_T_, tail mean error rate.

*Mental health.* Mental health included the subjective outcomes of depression, anxiety, stress, fatigue, sleep, and quality of life. The group who switched to A1PF milk had decreased anxiety over time compared to CON milk group (−0.61; *p* = 0.002; Table 4). While females experienced higher levels of anxiety compared to males (*p* = 0.004; Figure 6B), the beneficial effect of A1PF milk was observed in both sexes (milk type × sex interaction, *p* = 0.750). The A1PF milk group had marginally reduced subjective depression and stress compared to CON milk (−0.56, *p* = 0.022; and −0.70, *p* = 0.012, respectively; Table 4), with females experiencing higher levels of stress compared to males (*p* < 0.001; Figure 6C). Females also reported marginally higher fatigue compared to males (*p* = 0.040; Figure 6D), which was reduced with A1PF milk compared to CON milk (*p* = 0.001; Table 4). Milk type had no effect on sleep or quality of life and there was no interaction with sex in these outcomes.

#### Immune response

There was no effect of switching milk type on subjective or objective measures of immune response (Table 5). For salivary TNF-alpha, males showed a marginal decrease with A1PF milk which was not observed in females (*p* = 0.019; individual sex data not shown), and there was a marginal positive effect of age on salivary sIgA (*p* = 0.018), with an overall increase in sIgA with increasing age (data not shown). In subjective analyses, males reported a marginally higher level of immune status (*p* = 0.011).

**Table 5:** Immune-related outcomes for milk containing A2 β-casein only (A1PF milk) vs conventional milk containing both A1 and A2 β-caseins (CON milk).

Outcome variable	CON milk^1^			A1PF milk^1^			Difference over time (A1PF -CON)	*p*-values^2^				
	Day 0	Day 14	Day 28	Day 0	Day 14	Day 28		Milk type	Sex	Sex*milk type	Age	Usual milk intake
Salivary analysis												
IL-6 (pg/mL)	47.46 (16.8)	ND	48.40 (13.88)	53.96 (17.33)	ND	55.43 (14.32)	0.52 (18.04)	0.980	0.860	0.570	0.630	0.090
IL-1beta (pg/mL)	457.79 (53.33)	ND	516.89 (63.57)	451.85 (56.17)	ND	454.80 (66.96)	−56.16 (87.44)	0.520	0.160	0.380	0.650	0.770
sIgA (μg/mL)	278.11 (27.66)	ND	278.99 (26.01)	253.38 (28.92)	ND	279.58 (27.20)	25.33 (51.04)	0.620	0.140	0.230	0.018*	0.060
TNF-alpha (pg/mL)	30.86 (3.86)	ND	40.80 (3.67)	50.37 (3.98)	ND	51.79 (3.79)	−8.52 (6.35)	0.180	0.990	0.019*	0.084	0.360
Cortisol (μg/dL)	0.234 (0.019)	ND	0.278 (0.024)	0.252 (0.02)	ND	0.284 (0.026)	−0.011 (0.037)	0.760	0.053	0.180	0.140	0.880
Glutathione (total, nmol/L)	73.01 (5.48)	ND	94.81 (7.63)	60.02 (5.65)	ND	91.53 (5.65)	9.71 (14.34)	0.500	0.360	0.720	0.810	0.450
Subjective analysis												
Immune status^3^	7.71 (0.11)	9.07 (0.09)	9.17 (0.08)	7.28 (0.11)	8.80 (0.09)	8.98 (0.08)	0.25 (0.15)	0.160	0.011*	0.640	0.990	0.680
Respiratory symptoms^4^	0.548 (0.071)	0.546 (0.072)	0.439 (0.065)	0.730 (0.071)	0.674 (0.072)	0.595 (0.065)	−0.029 (103)	0.880	0.200	0.320	0.960	0.250

1 Values are means (SE).

2 *p*-values were derived using a repeated measures general linear model, with the Greenhouse–Geisser adjustment made for non-sphericity where appropriate. Significance was set at *P* < 0.01. There were no significant findings for immune related outcomes. Marginal significance was set between 0.01 and 0.05 and is indicated with an asterisk only.

3 Assessed via the Immune Status Questionnaire (ISQ).

4 Assessed via the Respiratory Symptoms Questionnaire (RSQ).

CON milk, milk containing both A1 and A2 β-casein proteins; A1PF, milk containing A2 β-casein only; IL, interleukin; ND, not determined; sIgA, secretory immunoglobulin A; TNF-alpha, tumour necrosis factor alpha.

#### Skin health

There was no effect of switching milk type on subjective skin health, measured by total skin complaints (Supplementary Table 5). Similarly, there were no effects of sex or usual milk intake, nor an interaction between milk type and any covariate.

## Discussion

This is the first study to explore the effects of reducing A1 β-casein intake via switching milk type from conventional milk containing both A1 and A2 β-casein proteins (CON milk) to A1-type β-casein free milk (A1PF milk) on brain, immune, gut, and skin-related outcomes in a healthy, free-living population. All necessary steps were taken to ensure both trial integrity and the reliability of results despite the open-label nature of the study design (36), contributing to an understanding of the role of A1PF milk in the diet of healthy individuals. While there was no observed impact on the gut microbiome, nor markers of immune function or skin health from reducing A1 β-casein in the diet, switching to A1PF milk produced a small decrease in stool consistency, and had marginal benefits for subjective GI symptoms and subjective cognitive function in females, compared to those who continued drinking CON milk. While these marginal benefits were small, limiting clinical relevance, an interesting finding warranting further exploration is the reduced anxiety, fatigue, depression, and stress, particularly in females, following a switch to A1PF milk. These findings suggest a potential novel role for A1PF milk in supporting the gut-brain axis in healthy adults, particularly regarding mood-related outcomes in females, that occurred without the complete elimination of A1-type β-casein from their diet. Results warrant confirmation and follow-up using a fully blinded trial design.

While the effects of an A1PF diet have traditionally been investigated in strictly controlled randomised trials, findings suggest that a complete elimination of A1-type β-casein may not be required to experience favourable effects for some health outcomes. RCTs and mechanistic data from animal research suggest that the ameliorating effects of removing A1-type β-casein on GI discomfort, inflammation, and cognitive impairment (10, 13, 15, 37), are due to, and require, the absence of the BCM7 opioid peptide. While A1-type β-casein is readily digested to produce BCM7, amino acid sequence differences in A2-type β-casein greatly reduce the likelihood of cleavage to produce BCM7 (5). In the BIGS Trial, the goal was not to completely eliminate all sources of A1-type β-casein from the diet (including cheese, yoghurt, ice-cream, and other sources), but to explore the effects of A1-type β-casein reduction on a wide range of health outcomes in a real-world setting, via a switch in milk type (as the major source of dairy in the Australian diet (38)). Given that A1PF cheese, yoghurt, ice-cream, and other milk products are not readily available for purchase, the switch in milk type tested in this study represents a likely scenario for individuals looking to trial the effects of A1PF milk on a range of health outcomes. Although no effects were found for GI inflammation, markers of immune response or for objective cognitive impairment, the decrease in stool consistency and possible benefits for GI comfort and subjective cognitive function in females that switched to A1PF milk are consistent with RCT evidence focused on the complete elimination of A1-type β-casein from the diet (10, 13, 15, 37). Further, for some markers, such as GSH, although not reaching significance, the direction of effect was consistent with that observed in highly controlled randomised studies (11). It is important to note that scores on the PROMIS Cognitive Function Scale have been shown to be influenced by negative affect scores (that is, negative mood scores) (39, 40). Therefore, an improvement in mood may lead to a higher subjective score of cognitive function, even if objective cognitive function has not changed. This phenomenon may help to explain the present results. In addition, in previous research (13), changes in objective cognitive function were linked to changes in inflammatory markers. As inflammation was not impacted by the switch in milk type in this study, a lack of effect on objective cognitive function might be expected. Overall, the findings indicate that there may be novel, dose-dependent, threshold-related, and sex-specific effects of A1-type β-casein reduction in healthy individuals, which warrant further investigation, including within a blinded and controlled setting involving the complete elimination of A1-type β-casein.

The potential beneficial effects of reducing A1-type β-casein on mood-related outcomes, particularly in females, is an important finding from within a real-world, pragmatic RCT and consistent with evidence implicating A1-type β-casein and BCM7 in the pathways associated with the aetiology of mental health disorders (41, 42). Since females also reported improvements in anxiety, fatigue, depression and stress after switching to A1PF milk, with reduced GI symptoms compared to males, there may be a sex-specific role for the gut-brain axis in mediating the mind and mood-related benefits of reducing A1-type β-casein in the diet. Differences in gut microbiome composition by milk type have been previously reported (5), with dysbiosis involving both Firmicutes phyla and the Clostridia class of gut microbiota, which belongs to the Firmicutes A phylum (43), being implicated in depression and generalised anxiety disorder (44). The BIGS Trial is the first study to report the effects of switching milk type on gut microbiome composition. While no effects on gut microbiome composition or diversity were found, a decrease in the Firmicutes A phylum was observed in those who reduced A1-type β-casein and thus a possible role of Firmicutes in adverse mood and/or digestive health cannot be dismissed. These findings have important implications for people with milk intolerance and other digestive health-related issues, in whom anxiety, depression, and a reduced quality of life are prevalent (45, 46), as well as individuals with clinical mental health diagnoses, who were excluded from this trial. For example, there may be a correlation between mood change and change in Firmicutes A abundance at the individual level, that is not observed for the overall population. While these results may also be explained, at least in part, by a placebo effect caused by the open label nature of the study (participants were aware of the type of milk they were drinking), further research to understand the role of the gut microbiome and its dominant bacterial phyla in modulating these effects is needed. In addition, while significant differences were observed in mood-related outcomes after a switch in milk type, scores remained within the level of “normal” according to DASS-21 threshold criteria (raw scores were doubled for threshold comparison) (47, 48). Given the healthy status of the recruited participant group, scores outside of the normal range for mood-related outcomes were not expected. Despite the limitations, there is an increased appreciation for the role of mental health in overall physiological health (49) and further research is required to investigate and better understand the therapeutic potential of A1PF milk in nutritional psychiatry, particularly for females.

Despite high quality RCT evidence demonstrating a relationship between the consumption of A1 β-casein and an adverse immune and inflammatory response (11, 13), the BIGS trial reported no effects of switching milk on systemic markers of immune function or inflammation. Findings might be explained by the continued presence of some A1-type β-casein in the diet, where a complete removal may be necessary to produce a significant effect. Alternatively, this study investigated the effects in a healthy population and perhaps the presence of a milk intolerance may mediate this effect on immune and inflammatory markers. In contrast to previous studies (10, 12–14), the BIGS trial was specifically designed to study usual consumers of conventional milk that were free of digestive health complaints. Similarly, no significant differences in the novel outcome of subjective skin health were reported in this study. However, as there is growing evidence linking the gut-brain axis to human health and disease (50, 51), and emerging research suggests a further link between gut and skin health (18), additional investigation is required to fully elucidate if A1-type β-casein plays a role in the modulation of skin health and related conditions.

Strengths of this study include the large national sample which increased statistical power and overall confidence in findings. The decentralised Australia-wide recruitment enabled findings to be generalisable to the healthy Australian adult population, while the pragmatic study design ensured findings were applicable to everyday life. The exploratory and open label nature of this trial may have attracted false-positive results because of multiple testing or the placebo effect; however, data are strengthened by a reduction in statistical significance to *p* < 0.01 during the analysis. There were some differences between some baseline measures of the CON and A1PF groups. While usual milk intake was higher in the CON milk arm of the total study group compared to the A1PF arm, this difference was small (46 g, 12% of a total intake of 398 g) and unlikely to be clinically significant. Similarly, while statistically different to the CON milk arm, the higher proportion of retired individuals in the A1PF arm of the total study group represents only 1.3% of the total study group and is unlikely to impact overall results. Lastly, recruitment of healthy individuals free of digestive health complaints and regularly consuming conventional milk may indicate a reduced likelihood for BCM7 intolerance. It is possible that a background of intolerance is necessary to detect differences in the gut microbiome, as well as inflammatory markers, objective cognitive measures, skin health, and more. However, evidence regarding the health effects of milk type in healthy individuals is necessary for population-based dietary recommendations, making a significant contribution to current understanding in this area.

## Conclusion

This study provides real-world evidence that reducing A1 β-casein intake by switching from conventional milk to A1PF milk may provide a novel benefit for mood and mental health-related outcomes in healthy free-living individuals, especially females, with potential links to the gut-brain axis. Although microbiome composition and diversity did not appear to directly mediate these effects, the role of the gut microbiota as a central tenet in the interrelated nature of physical and mental health requires further investigation. These findings make an important contribution to understanding the impact of a reduction of A1-type β-casein, by a simple switch in milk type, on health in the general population and suggest a role for A1PF milk in supporting mood and mental health in healthy individuals. While these effects occurred without the need for the complete elimination of A1 β-casein, greater effects may occur when all A1-type β-casein is removed from the diet. Due to the open-label design of the study, replication of this research in fully blinded trials and on specific population groups, including milk-intolerant individuals, females with low mood, and those experiencing GI conditions, is warranted.

## Data Availability

The raw data supporting the conclusions of this article will be made available by the authors without undue reservation.

## Footnotes

^1^ https://intake24.co.uk/

^2^ http://gtdb.ecogenomic.org/

^3^ https://scit.neurotest.com
