# Supplementary material for: A1 protein free milk benefits mood and subjective cognition in free-living Australian adults: a pragmatic, exploratory, open label randomised controlled trial
Source: Front Nutr. 2025 Jun 3;12:1579986. doi: 10.3389/fnut.2025.1579986 (PMC12170308; doi:10.3389/fnut.2025.1579986)
Supplement: Supplementary file 1 [file Table_1.docx]

Supplementary Material

**Supplementary Table 1.** Description and validity of the subjective/self-reported questionnaires used in the BIGS Trial.

| Self-reported questionnaire | Description | Validity | Outcomes measured |
| --- | --- | --- | --- |
| Gastrointestinal symptom rating scale (GSRS) | The GSRS total score ranges from 0-40 with higher scores representing more severe symptoms, and each sub-score for each of the domain ranges from 0-10. Total and subcategory scores will be normalised to be reported on a scale of 0 (worst symptoms) to 100 (no symptoms). There are 15 symptom specific items each graded on a 7-point Likert Scale | Shown as valid and reliable in a range of gastrointestinal functional disorders or diseases (1-3). Demonstrated use in multiple Australian research settings (4, 5). | 1) Overall gastrointestinal symptoms, 2) Abdominal pain sub-score, 3) Reflux sub-score, 4) Diarrhea sub-score, 5) Indigestion sub-score, 6) Constipation sub-score. |
| Bristol Stool Chart (BSC) | The BSC rates tools on a scale from “type 1” (stool characteristic of constipation) through to “type 7” (stool characteristic of diarrhea). Score can be reported categorically or continuously. Participants will be asked to rate their last bowel motion according to the Bristol Stool Chart (BSC), with the BSC picture added to the electronic survey for reference. | Demonstrated validity and reliability (6); widely used in clinical practice and research settings (7). | Stool consistency |
| Immune Status Questionnaire (ISQ) | The ISQ rates immune health-related complaints in the past 12 months from ‘Never’ to ‘Almost always’. The test has three domains, with 7 items corresponding with each domain, and the average completion time is 1-minute. | Demonstrated reliability and validity to assess perceived immune status in clinical and research settings (8). | Immune status |
| PROMIS-SF Cognitive Function 6a​ | The PROMIS-SF Cognitive Function 6a rates statements about cognitive function in the past 7 days on a scale from 5 ‘Never’ to 1 ‘Very often (several times a day)’. Scores can be reported categorically or continuously. The test has six items, and the average completion time is <1-minute. | Demonstrated validity to assess cognitive function in clinical and research settings (9, 10). | Cognitive function |
| PROMIS-SF Fatigue 4​ | The PROMIS-SF Fatigue 4a rates statements about fatigue during/in the past 7 days on a scale from 1 ‘Not at all’ to 5 ‘Very much’. Scores can be reported categorically or continuously. The test has four items, and the average completion time is <1-minute. | Demonstrated validity to assess fatigue in clinical and research settings (11, 12). | Fatigue |
| Skin morbidity questionnaire | Self-reported questionnaire to assess skin morbidity and dermatologic life-quality, including skin complaints and variables such as demographic, psychosocial, general health, dermatologic, and life-quality items (13). Higher skin morbidity scores represent a greater presence of skin disease (13). | Validated for use in the general adult population (14). | Total skin complaints |
| Depression, Anxiety and Stress Scale-21 Items (DASS 21) | The DASS-21 rates depression, anxiety, and stress on a scale from 0 ‘Did not apply to me at all’ up to 3 ‘Applied to me very much or most of the time’. The test has three domains, with 7 items corresponding with each domain, and the average completion time is 3-minutes. | The DASS-21 is applicable in clinical, medical, and research settings and has been validated in adult populations (15). | Mental health; depression, anxiety, and stress. Scores presented as raw scores. |
| Respiratory symptoms questionnaire (RSQ) | The RSQ rates respiratory symptoms over the last 4 weeks on a 5-point Likert scale. Scores can be reported categorically. The test has 4 items, and the average completion time is <1-minute. | Demonstrated validity to assess respiratory symptoms, including asthma, in clinical and research settings (16). | Respiratory symptoms; shortness of breath, wheezing, coughing, and chest tightness. |
| World Health Organization – Five Well-being Index (WHO-5) | The WHO-5 rates statements about subjective wellbeing in the past two weeks on a scale from 5 ‘All of the time’ to 0 ‘At no time’. Scores can be reported categorically or continuously. The test has 5 items, and the average completion time is 1-minute. | Demonstrated validity and reliability for psychometric evaluation in variety of adult populations (17-19). One of the most widely used questionnaires to asses subjective psychological wellbeing. | General overall wellbeing |
| EuroQoL visual analogue scale (EQ-VAS) | Visual analogue scale from 0 to 100 that measures subjective overall health on the current day (20). A higher number (maximum 100) is indicative of better subjective overall current health (20). | Reliable for evaluating health-related quality of life in a primary care setting (21). | Overall current health |
| PROMIS-SF Sleep Disturbance 6a | The PROMIS-SF Sleep Disturbance 6a rates statements about i) sleep quality in the past 7 days on a scale from 5 ‘Very poor’ to 1 ‘Very good’, and ii) sleep disturbances in the past 7 days on a scale from ‘Not at all’ to ‘Very much’. Scores can be reported categorically or continuously. The test has 6 items, and the average completion time is 1-minute. | Demonstrated accuracy and reliability in assessment of sleep quality in adults (22, 23). | Sleep quality |

**Supplementary Table 2.** Differential abundance at the phylum level within and between study groups (adjusted data^1^).

| Taxon | CON - within trial | | | | | | A1PF- within trial | | | | | | P value^2^ - between trials (treatment effect) | | | |
| --- | --- | --- | --- | --- | --- | --- | --- | --- | --- | --- | --- | --- | --- | --- | --- | --- |
|  | Mean (SD) | | P value^2^ | | | | Mean (SD) | | P value^2^ | | | |  |  |  |  |
|  | Pre | Post | sqrt | FDR sqrt | clr | FDR  clr | Pre | Post | sqrt | FDR sqrt | clr | FDR  clr | sqrt | FDR sqrt | clr | FDR  clr |
| Actino-bacteriota | 9  (10) | 8.6  (8.5) | 0.810 | 0.950 | 0.910 | 0.910 | 8.1  (8.5) | 9.2  (10) | 0.099 | 0.490 | 0.600 | 0.600 | 0.230 | 0.480 | 0.620 | 0.840 |
| Bacteroidota | 20  (10) | 21  (8.7) | 0.330 | 0.920 | 0.610 | 0.650 | 20  (8.7) | 19  (8) | 0.790 | 0.820 | 0.190 | 0.540 | 0.360 | 0.620 | 0.210 | 0.710 |
| Campylo-bacterota | 0.02  (0.11) | 0.046  (0.15) | 0.028 | 0.200 | 0.042 | 0.590 | 0.015  (0.054) | 0.052 (0.23) | 0.067 | 0.490 | 0.130 | 0.540 | 0.960 | 0.960 | 0.880 | 0.940 |
| Cyanobacteria | 0.11  (0.55) | 0.12  (0.5) | 0.910 | 0.950 | 0.540 | 0.650 | 0.14  (0.45) | 0.18 (0.62) | 0.340 | 0.700 | 0.530 | 0.590 | 0.480 | 0.630 | 0.410 | 0.780 |
| Desulfo-bacterota A | 0.16  (0.16) | 0.18  (0.21) | 0.099 | 0.350 | 0.150 | 0.590 | 0.16  (0.18) | 0.16 (0.17) | 0.650 | 0.790 | 0.460 | 0.580 | 0.400 | 0.620 | 0.640 | 0.840 |
| Euryarchaeota | 0.2  (0.53) | 0.2  (0.53) | 0.520 | 0.930 | 0.280 | 0.590 | 0.22  (0.72) | 0.23 (0.68) | 0.680 | 0.790 | 0.330 | 0.580 | 0.900 | 0.960 | 0.940 | 0.940 |
| Firmicutes | 3.1  (2.5) | 3.5  (4.1) | 0.610 | 0.930 | 0.300 | 0.590 | 3.9  (3.8) | 3.9  (3.7) | 0.820 | 0.820 | 0.220 | 0.540 | 0.590 | 0.660 | 0.820 | 0.940 |
| Firmicutes A | 47  (13) | 48  (12) | 0.820 | 0.950 | 0.240 | 0.590 | 49  (11) | 46  (14) | 0.009 | 0.140 | 0.003 | **0.039** | 0.027 | 0.230 | 0.050 | 0.640 |
| Firmicutes B | 0.012 (0.06) | 0.001  (0.04) | 0.950 | 0.950 | 0.420 | 0.590 | 0.019  (0.079) | 0.019 (0.077) | 0.370 | 0.700 | 0.410 | 0.580 | 0.500 | 0.630 | 0.280 | 0.740 |
| Firmicutes C | 2.1  (3.2) | 1.9  (2.2) | 0.460 | 0.930 | 0.360 | 0.590 | 1.8  (2.8) | 1.9  (3.2) | 0.260 | 0.640 | 0.300 | 0.580 | 0.190 | 0.470 | 0.140 | 0.710 |
| Firmicutes G | ND | ND | ND | ND | ND | ND | 0.001  (0.004) | 0.001 (0.003) | 0.130 | 0.490 | 0.023 | 0.170 | 0.190 | 0.470 | 0.640 | 0.840 |
| Fuso-bacteriota | 0.035 (0.018) | 0.03  (0.13) | 0.530 | 0.930 | 0.410 | 0.590 | 0.11  (0.51) | 0.13 (0.67) | 0.490 | 0.790 | 0.550 | 0.590 | 0.520 | 0.630 | 0.350 | 0.740 |
| Proteo-bacteria | 2.7  (7.7) | 2.1  (3.6) | 0.670 | 0.930 | 0.560 | 0.650 | 2.3  (3.4) | 3.8  (11) | 0.190 | 0.580 | 0.440 | 0.580 | 0.190 | 0.470 | 0.320 | 0.740 |
| Synergistota | 0.0003 (0.003) | 0.004  (0.017) | 0.006 | 0.083 | 0.320 | 0.590 | 0.002  (0.011) | 0.001 (0.006) | 0.610 | 0.790 | 0.140 | 0.540 | 0.015 | 0.230 | 0.075 | 0.640 |
| Verruco-microbiota | 0.28  (0.65) | 0.5  (1.5) | 0.057 | 0.260 | 0.260 | 0.590 | 0.4  (1.1) | 0.52 (2.2) | 0.590 | 0.790 | 0.420 | 0.580 | 0.400 | 0.620 | 0.860 | 0.940 |

^1^ Data were adjusted for milk intake, sex, age, dietary fibre intake, and the presence of a health condition. ^2^ P values are presented for square root (sqrt) and centre-log ratio (clr) transformations, both uncorrected and following correction for false discovery rate (FDR). P values remaining significant after FDR correction are indicated in bold. ND, not determined. CON, conventional milk containing both A1 and A2 β-casein proteins; A1PF milk, milk containing A2 β-casein only.

**Supplementary Table 3.** Top 20 differentially abundant families, genera, and species between milk types (adjusted data^1^).

| Taxon | P value^2^ | | | |
| --- | --- | --- | --- | --- |
|  | **sqrt** | **FDR sqrt** | **clr** | **FDR clr** |
| Family | | | | |
| Aerococcaceae | 0.040 | 0.760 | 0.040 | 0.750 |
| Brevibacteriaceae | 0.079 | 0.760 | 0.077 | 0.750 |
| CAG-239 | 0.410 | 0.850 | 0.043 | 0.750 |
| CAG-313 | 0.120 | 0.760 | 0.340 | 0.820 |
| Filifactoraceae | 0.089 | 0.760 | 0.210 | 0.820 |
| Gemellaceae | 0.200 | 0.760 | 0.100 | 0.750 |
| Lachnospiraceae | 0.190 | 0.760 | 0.065 | 0.750 |
| Megasphaeraceae | 0.088 | 0.760 | 0.066 | 0.750 |
| Microbacteriaceae | 0.110 | 0.760 | 0.170 | 0.820 |
| Monoglobaceae | 0.500 | 0.920 | 0.089 | 0.750 |
| Propionibacteriaceae | 0.120 | 0.760 | 0.061 | 0.750 |
| QAND01 | 0.059 | 0.760 | 0.051 | 0.750 |
| Ruminococcaceae | 0.100 | 0.760 | 0.080 | 0.750 |
| Selenomonadaceae | 0.093 | 0.760 | 0.054 | 0.750 |
| Staphylococcaceae | 0.091 | 0.760 | 0.130 | 0.820 |
| Succinivibrionaceae | 0.230 | 0.760 | 0.100 | 0.750 |
| Synergistaceae | 0.009 | 0.760 | 0.044 | 0.750 |
| UBA1242 | 0.110 | 0.760 | 0.053 | 0.750 |
| UBA1390 | 0.170 | 0.760 | 0.110 | 0.750 |
| UBA932 | 0.056 | 0.760 | 0.054 | 0.750 |
| Genus | | | | |
| Bacteroides | 0.039 | 0.920 | 0.280 | 0.890 |
| Blautia_A | 0.082 | 0.920 | 0.042 | 0.890 |
| Butyricicoccaceae MIC8222 | 0.003 | 0.880 | 0.006 | 0.730 |
| CAG-177 | 0.045 | 0.920 | 0.022 | 0.890 |
| CAG-245 | 0.010 | 0.880 | 0.012 | 0.740 |
| CAG-521 | 0.160 | 0.920 | 0.044 | 0.890 |
| CAG-74 MIC9650 | 0.021 | 0.920 | 0.034 | 0.890 |
| CAG-74 MIC9837 | 0.017 | 0.880 | 0.008 | 0.730 |
| Cloacibacillus | 0.012 | 0.880 | 0.038 | 0.890 |
| Facklamia | 0.017 | 0.880 | 0.012 | 0.740 |
| Faecalicatena | 0.061 | 0.920 | 0.007 | 0.730 |
| Firm-07 | 0.022 | 0.920 | 0.046 | 0.890 |
| Gemmiger | 0.081 | 0.920 | 0.043 | 0.890 |
| Gordonibacter | 0.014 | 0.880 | 0.025 | 0.890 |
| Lactococcus | 0.006 | 0.880 | 0.009 | 0.730 |
| Megasphaera | 0.058 | 0.920 | 0.039 | 0.890 |
| Olsenella_E | 0.013 | 0.880 | 0.009 | 0.730 |
| Ruminiclostridium_C | 0.100 | 0.920 | 0.024 | 0.890 |
| UBA1691 | 0.052 | 0.920 | 0.026 | 0.890 |
| UBA5446 | 0.040 | 0.920 | 0.051 | 0.890 |
| Species | | | | |
| s__Alistipes_A ihumii | 0.015 | 0.860 | 0.009 | 0.860 |
| s__Blautia_A MIC9663 | 0.012 | 0.860 | 0.004 | 0.860 |
| s__Blautia_A sp900066165 | 0.056 | 0.860 | 0.005 | 0.860 |
| s__Butyricicoccaceae MIC8222 | 0.003 | 0.860 | 0.005 | 0.860 |
| s__CAG-74 MIC9837 | 0.017 | 0.860 | 0.010 | 0.860 |
| s__Clostridium_M sp001517625 | 0.034 | 0.860 | 0.012 | 0.880 |
| s__Desulfovibrio fairfieldensis | 0.016 | 0.860 | 0.010 | 0.860 |
| s__Dorea MIC6991 | 0.015 | 0.860 | 0.016 | 0.880 |
| s__Dorea sp900066765 | 0.006 | 0.860 | 0.008 | 0.860 |
| s__Faecalibacterium prausnitzii_C | 0.006 | 0.860 | 0.0002 | 0.230 |
| s__Faecalibacterium prausnitzii_J | 0.036 | 0.860 | 0.008 | 0.860 |
| s__Faecalicatena faecis | 0.020 | 0.860 | 0.017 | 0.880 |
| s__Finegoldia MIC6997 | 0.014 | 0.860 | 0.017 | 0.880 |
| s__Gemmiger MIC9530 | 0.002 | 0.860 | 0.001 | 0.500 |
| s__Intestinimonas butyriciproducens | 0.011 | 0.860 | 0.018 | 0.880 |
| s__Lachnospira eligens_B | 0.081 | 0.860 | 0.008 | 0.860 |
| s__Olsenella_E MIC6865 | 0.017 | 0.860 | 0.017 | 0.880 |
| s__Prevotella disiens | 0.014 | 0.860 | 0.006 | 0.860 |
| s__Ruminiclostridium_C sp000435295 | 0.050 | 0.860 | 0.014 | 0.880 |
| s__Streptococcus parasanguinis_B | 0.024 | 0.860 | 0.015 | 0.880 |

^1^ Data were adjusted for milk intake, sex, age, dietary fibre intake, and the presence of a health condition. ^2^ P values are presented for square root (sqrt) and centre-log ratio (clr) transformations, both uncorrected and following correction for false discovery rate (FDR).

**Supplementary Table 4.** Top 20 differentially abundant functions between milk types (adjusted data^1^), based on MetaCyc groups, MetaCyc pathways, enzyme commission numbers, and the membrane transporter classification database (TCDB).

| MetaCyc Pathway | MetaCyc Group | Enzymes (EC number) | Membrane transport proteins  (TCDB database) |
| --- | --- | --- | --- |
| L-lysine biosynthesis I | Acetyl Co-A biosynthesis | Mannitol-1-phosphate 5-dehydrogenase | OmpA family protein |
| D-gluconate degradation | Alcohol degradation | 3(or 17)- b-hydroxysteroid dehydrogenase | Outer membrane usher protein htrE |
| Mannitol degradation I | Aldehyde degradation | Long-chain-alcohol dehydrogenase | Putative uncharacterized protein |
| O-antigen building blocks biosynthesis (*E. coli*) | Aromatic compound Biosynthesis | Aldehyde dehydrogenase (FAD-independent) | Colicin K |
| Pentose phosphate pathway | Carbohydrate biosynthesis | Anhydrotetracycline 6-monooxygenase | Holin, BlyA family |
| Superpathway of pyrimidine ribonucleotides de novo biosynthesis | Carbohydrate degradation | D-proline reductase | Sugar efflux transporter A |
| Phenylacetate degradation I aerobic | Cell structure biosynthesis | 3-methyl-2-oxobutanoate hydroxymethyltransferase | Transporter, major facilitator family protein |
| Fructoselysine and psicoselysine degradation | Cofactor prosthetic group electron carrier and vitamin biosynthesis | Glycine amidinotransferase | Putative fructoselysine transporter FrlA |
| Sucrose degradation IV sucrose phosphorylase | Degradation utilization assimilation other | Scyllo-inosamine-4-phosphate amidinotransferase | Inner membrane protein YdcZ |
| Pyruvate fermentation to S lactate | Fermentation | N-acetylneuraminate synthase | Tellurite resistance protein |
| UDP N acetyl D galactosamine biosynthesis I | Glycan biosynthesis | Adenosyl-fluoride synthase | Sodium/proline symporter |
| Hydroxyphenylpyruvate biosynthesis | Hormone biosynthesis | N-acylneuraminate cytidylyltransferase | Trk system potassium uptake protein trkG |
| CMP N acetylneuraminate biosynthesis II bacteria | Inorganic nutrient metabolism | Cyclin-dependent kinase | L-tartrate:succinate antiporter |
| S methyl 5 thioadenosine degradation III | Nucleoside and nucleotide Biosynthesis | UDP-N-acetylglucosamine 2-epimerase | Glutamine transporter GlnQP |
| Protein N glycosylation bacterial | Pentose phosphate pathways | Allantoinase | Uptake system for glycine-betaine (high affinity) and proline (low affinity) |
| Inosine 5 phosphate biosynthesis III | Protein modification | P-type Ag(+) transporter | Not listed |
| UDP α D glucose biosynthesis I | Reactive oxygen species degradation | UDP-N-acetylglucosamine 4,6-dehydratase | Ag^+^-ATPase (efflux) |
| Atromentin biosynthesis | Respiration | S-(hydroxymethyl)glutathione synthase | Flagellar protein export system. |
| UDP yelosamine biosynthesis | Secondary metabolite biosynthesis | Phosphatidylinositol diacylglycerol-lyase | Putative uncharacterized protein |
| Glycine degradation Stickland reaction | Unclassified pathways | Fructoselysine 3-epimerase | Inner membrane protein yhaI |

^1^ Data were adjusted for milk intake, sex, age, dietary fibre intake, and the presence of a health condition. Ag, silver; CMP, cytidine monophosphate; EC, Enzyme Commission number; FAD, flavin a vbidenine dinucleotide; TCDB, Transporter Classification Database; UDP, uridine diphosphate

**Supplementary Table 5.** Subjective skin-related health outcomes for milk containing A2 β-casein only (A1 protein free milk) vs conventional milk containing both A1 and A2 β-caseins (A1/A2 milk).

| Outcome variable | CON milk | | | A1PF milk | | | Difference over time  (A1PF-CON) | P-value^1^ | | | | |
| --- | --- | --- | --- | --- | --- | --- | --- | --- | --- | --- | --- | --- |
|  | Day 0 | Day 14 | Day 28 | Day 0 | Day 14 | Day 28 |  | Milk type | Sex | Sex*milk type | Usual milk intake | Age |
| Skin complaints^2^ | ND | 0.677 (0.04) | 0.691 (0.04) | ND | 0.762 (0.04) | 0.743 (0.04) | -0.033 | 0.462 | 0.964 | 0.607 | 0.582 | 0.987 |

^1^ P-values were derived using a repeated measures general linear model, with the Greenhouse-Geisser adjustment made for non-sphericity where appropriate. Significance was set at P<0.01. ^2^ Values are means (SE). CON, conventional milk containing both A1 and A2 β-casein proteins; A1PF milk, milk containing A2 β-casein only; ND, not determined

**References**

1. Revicki DA, Wood M, Wiklund I, Crawley J. Reliability and validity of the Gastrointestinal Symptom Rating Scale in patients with gastroesophageal reflux disease. Quality of life research. 1997;7(1):75-83.

2. Kulich KR, Madisch A, Pacini F, Piqué JM, Regula J, Van Rensburg CJ, et al. Reliability and validity of the Gastrointestinal Symptom Rating Scale (GSRS) and Quality of Life in Reflux and Dyspepsia (QOLRAD) questionnaire in dyspepsia: a six-country study. Health and quality of life outcomes. 2008;6(1):1-12.

3. Ljótsson B, Jones M, Talley NJ, Kjellström L, Agréus L, Andreasson A. Discriminant and convergent validity of the GSRS-IBS symptom severity measure for irritable bowel syndrome: A population study. United European gastroenterology journal. 2020;8(3):284-92.

4. ACTRN12620000251921p Bariatric Body Composition Study: A prospective cohort study comparing post-procedural body composition and bone density changes via dual-energy X-ray absorptiometry (DXA) for patients electing bariatric surgery in Australia <https://anzctr.org.au/>: ANZCTR; 2020 [

5. ACTRN12616001378415 Dietary management of uncomplicated diverticulitis: what is the effect of inpatient dietary restriction and discharge diet prescription on length of stay, recurrence and patient outcomes in patients admitted to an acute-care hospital in South-East Queensland, Australia? <https://anzctr.org.au/>: ANZCTR; 2016 [

6. Ohno H, Murakami H, Tanisawa K, Konishi K, Miyachi M. Validity of an observational assessment tool for multifaceted evaluation of faecal condition. Scientific reports. 2019;9(1):1-9.

7. Cingolani A, Paduano D, Vecchiarelli V, Demelas M, Corrias PT, Casula L, Usai P. Feasibility of low fermentable oligosaccharide, disaccharide, monosaccharide, and polyol diet and its effects on quality of life in an Italian cohort. Nutrients. 2020;12(3):716.

8. Wilod Versprille J, van de Loo J, Mackus M, Arnoldy L, T ALS, Vermeulen SA, et al. Development and Validation of the Immune Status Questionnaire (ISQ). Int J Environ Res Public Health. 2019;16(23).

9. Henneghan AM, Van Dyk K, Zhou X, Moore RC, Root JC, Ahles TA, et al. Validating the PROMIS cognitive function short form in cancer survivors. Breast Cancer Res Treat. 2023;201(1):139-45.

10. Becker H, Stuifbergen A, Lee H, Kullberg V. Reliability and Validity of PROMIS Cognitive Abilities and Cognitive Concerns Scales Among People with Multiple Sclerosis. International journal of MS care. 2014;16:1-8.

11. Bingham Iii CO, Gutierrez AK, Butanis A, Bykerk VP, Curtis JR, Leong A, et al. PROMIS Fatigue short forms are reliable and valid in adults with rheumatoid arthritis. J Patient Rep Outcomes. 2019;3(1):14.

12. Terwee CB, Elsman EB, Roorda LD. Towards standardization of fatigue measurement: Psychometric properties and reference values of the PROMIS Fatigue item bank in the Dutch general population. Research Methods in Medicine & Health Sciences. 2022;3(3):86-98.

13. Dalgard F, Svensson A, Holm J, Sundby J. Self-reported skin morbidity among adults: associations with quality of life and general health in a Norwegian survey. J Investig Dermatol Symp Proc. 2004;9(2):120-5.

14. Dalgard F, Svensson A, Holm J, Sundby J. Self-reported skin complaints: validation of a questionnaire for population surveys. Br J Dermatol. 2003;149(4):794-800.

15. Ng F, Trauer T, Dodd S, Callaly T, Campbell S, Berk M. The validity of the 21-item version of the Depression Anxiety Stress Scales as a routine clinical outcome measure. Acta Neuropsychiatr. 2007;19(5):304-10.

16. Karlsson N, Atkinson MJ, Müllerová H, Alacqua M, Keen C, Hughes R, et al. Validation of a diagnosis-agnostic symptom questionnaire for asthma and/or COPD. ERJ Open Res. 2021;7(1).

17. Halliday JA, Hendrieckx C, Busija L, Browne JL, Nefs G, Pouwer F, Speight J. Validation of the WHO-5 as a first-step screening instrument for depression in adults with diabetes: Results from Diabetes MILES - Australia. Diabetes Res Clin Pract. 2017;132:27-35.

18. Fung SF, Kong CYW, Liu YM, Huang Q, Xiong Z, Jiang Z, et al. Validity and Psychometric Evaluation of the Chinese Version of the 5-Item WHO Well-Being Index. Front Public Health. 2022;10:872436.

19. Cosma A, Költő A, Chzhen Y, Kleszczewska D, Kalman M, Martin G. Measurement Invariance of the WHO-5 Well-Being Index: Evidence from 15 European Countries. Int J Environ Res Public Health. 2022;19(16).

20. Gusi N, Olivares PR, Rajendram R. The EQ-5D Health-Related Quality of Life Questionnaire. In: Preedy VR, Watson RR, editors. Handbook of Disease Burdens and Quality of Life Measures. New York, NY: Springer New York; 2010. p. 87-99.

21. Xu RH, Sun R, Tian L, Cheung AW-l, Wong EL. Health-related quality of life in primary care patients: a comparison between EQ-5D-5L utility score and EQ-visual analogue scale. Health and Quality of Life Outcomes. 2024;22(1):2.

22. Full KM, Malhotra A, Crist K, Moran K, Kerr J. Assessing psychometric properties of the PROMIS Sleep Disturbance Scale in older adults in independent-living and continuing care retirement communities. Sleep Health. 2019;5(1):18-22.

23. Chimenti RL, Rakel BA, Dailey DL, Vance CGT, Zimmerman MB, Geasland KM, et al. Test-Retest Reliability and Responsiveness of PROMIS Sleep Short Forms Within an RCT in Women With Fibromyalgia. Front Pain Res (Lausanne). 2021;2:682072.
